# 348. Impact of Substance Use Disorders on Outcomes of Medically Insured Persons Receiving Multiweek Outpatient Parenteral Antimicrobial Therapy

**DOI:** 10.1093/ofid/ofad500.419

**Published:** 2023-11-27

**Authors:** Daniel Rogers, Daniel B Chastain, Lingyu Zhao, Duna Zhan, Xianyan Chen

**Affiliations:** University of Georgia/Emory Saint Joseph's Hospital, Lawrenceville, Georgia; University of Georgia College of Pharmacy, Albany, Georgia; University of Georgia, Athens, Georgia; dunaz@uga.edu, Athens, Georgia; UGA Franklin College of Arts and Sciences, Athens, Georgia

## Abstract

**Background:**

Catheter complications, misuse, nonadherence, safety, and legal concerns often prohibit persons with a history of substance use disorder (SUD) from receiving outpatient parenteral antimicrobial therapy (OPAT). However, single center studies in the U.S. found similar rates of readmission and adverse events among patients receiving OPAT regardless of SUD history. As such, we compared outcomes and complications between patients with and without a history of SUD who were treated with multiweek OPAT using claims data from multiple years across the U.S.

**Methods:**

We used data from the IBM MarketScan Databases to identify patients who were ≥ 18 years and treated with intravenous vancomycin, daptomycin, nafcillin, oxacillin, or cefazolin for ≥ 7 days after hospital discharge between July 1, 2016 and July 31, 2020. Those with a history of end stage renal disease or *Clostridioides difficile* infection were excluded. Patients were separated into two cohorts based on a history of SUD using ICD-10-CM diagnosis codes prior to hospital discharge. The rates of adverse events including drug overdose and catheter complications during OPAT, all-cause readmissions within 90 days after completion of OPAT, and infection-related readmissions within 90 days after completing OPAT were compared between groups.

**Results:**

Of 5903 patients included, 18% (n = 1058) had a history of SUD. Those with a history of SUD were younger than individuals without a history of SUD (47 ± 12 vs 53 ± 15 years, P < 0.0001) and there were fewer male patients in the SUD group (52% vs 56%, P = 0.02). Type 2 diabetes mellitus (43% vs 52%, P < 0.0001), arrhythmias (33% vs 31%, P = 0.152), and depression (49% vs 30%, P < 0.0001) were most common among both groups. More patients in the SUD group experienced at least one adverse event during OPAT (8% vs 5%, P = 0.004). All-cause readmissions and infection-related readmissions within 90 days after completion of OPAT were more common among patients with a history of SUD (40% vs 32%, P < 0.0001 and 27% vs 22%, P = 0.02, respectively).

**Conclusion:**

Patients with a history of SUD experienced higher rates of adverse events during OPAT and were more likely to require readmission for any reason, including readmissions due to infection, within 90 days after completion of OPAT.

**Disclosures:**

**All Authors**: No reported disclosures

